# Supplementary figures and images for: Inhibition of Chikungunya virus nsP2 protease in vitro by scorpion venom peptide pantinin-1
Source: PLoS One. 2026 Apr 9;21(4):e0346930. doi: 10.1371/journal.pone.0346930 (PMC13065029; doi:10.1371/journal.pone.0346930)

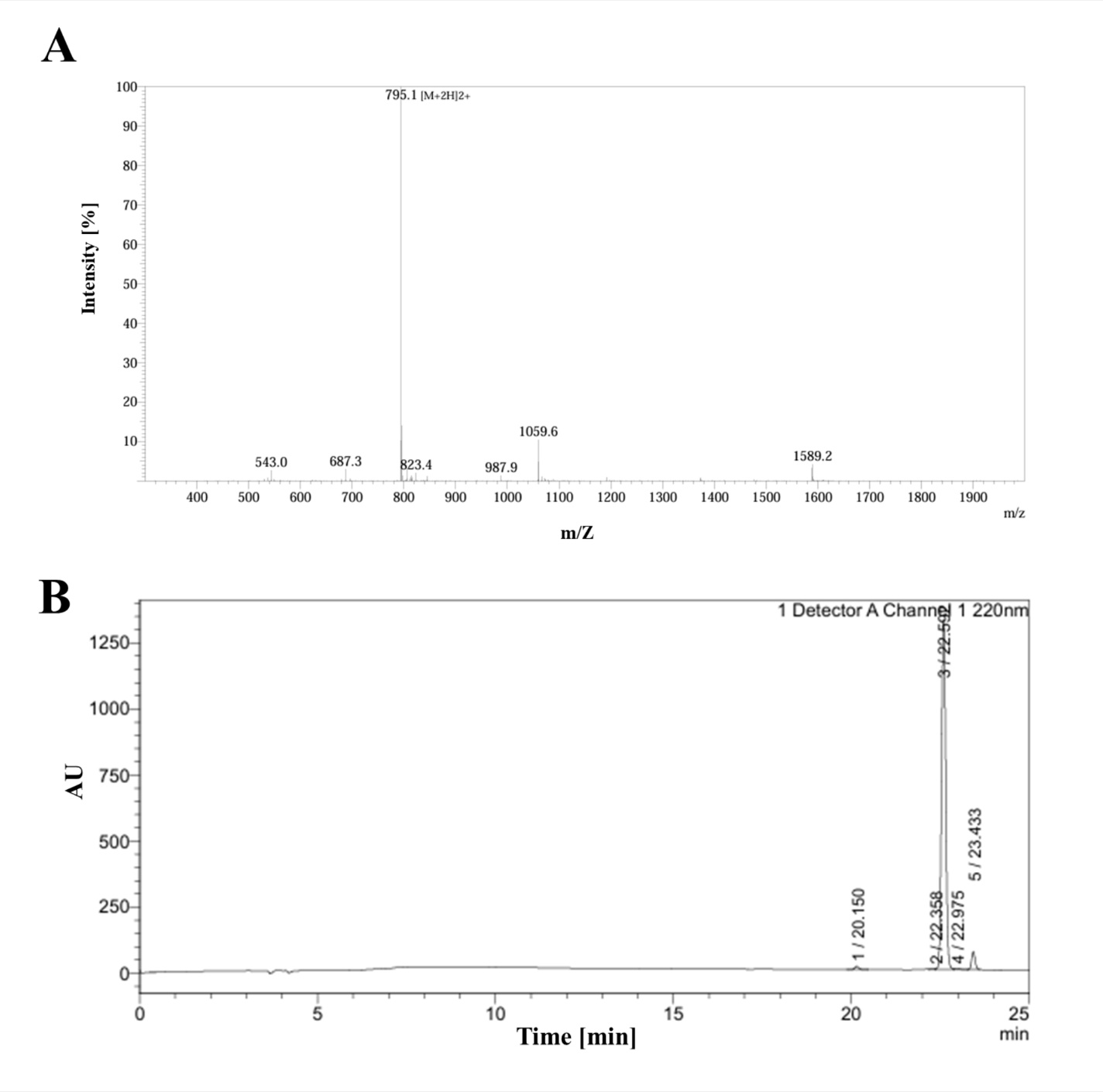

Supplement: S2 Fig — (A) Mass spectrometry data provided by GenScript Biotech (Netherlands) confirming the molecular weight and purity of the synthesized Pantinin-1 peptide. (B) HPLC chromatogram showing the retention time and purity profile of pantinin-1. (TIF) [file pone.0346930.s002.tif]

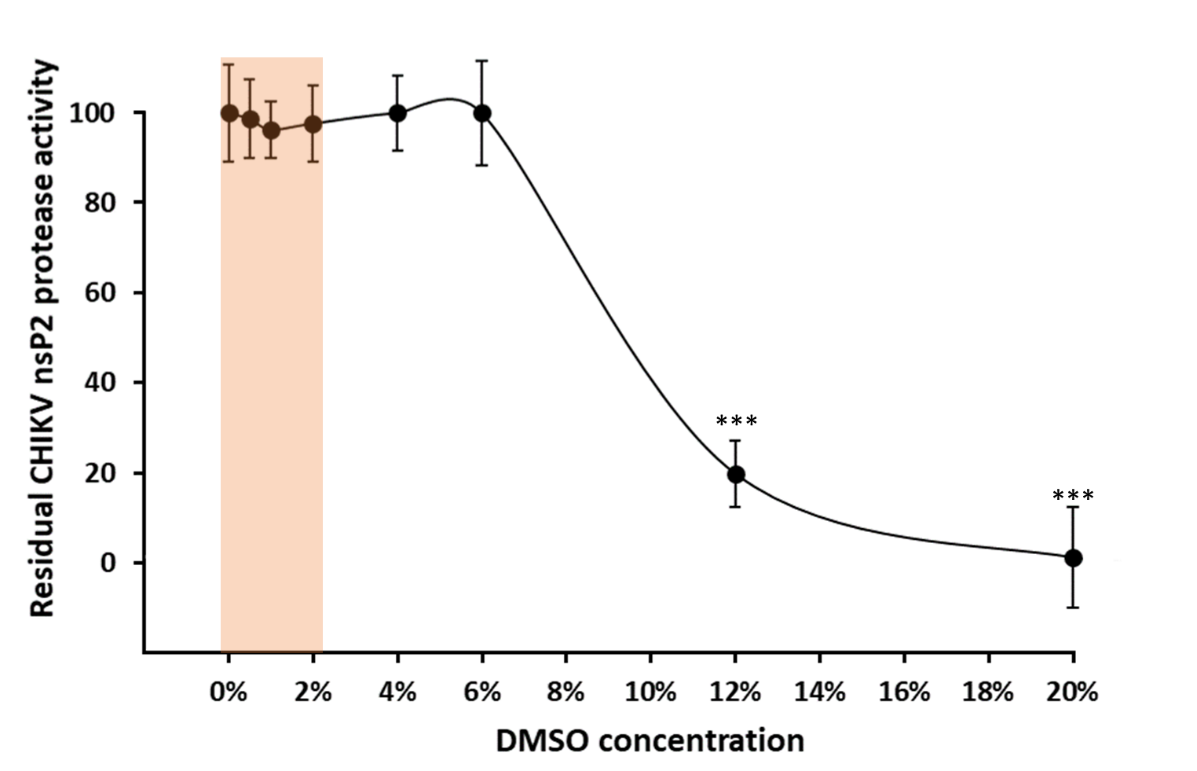

Supplement: S3 Fig — A control experiment was performed to assess the influence of DMSO on protease activity at concentrations of up to 20%. No significant changes in protease activity were detected at DMSO concentrations ≤6%, indicating that DMSO is an appropriate solvent within this range. The rose box denotes the DMSO concentration range applied in the inhibition assays. Differences among groups were evaluated using one-way ANOVA with Tukey’s multiple-comparison test. Significant differences relative to the control (0 µM inhibitor) are indicated by asterisks (***, p < 0.001). Values represent mean ± SD (n = 3). (TIF) [file pone.0346930.s003.tif]

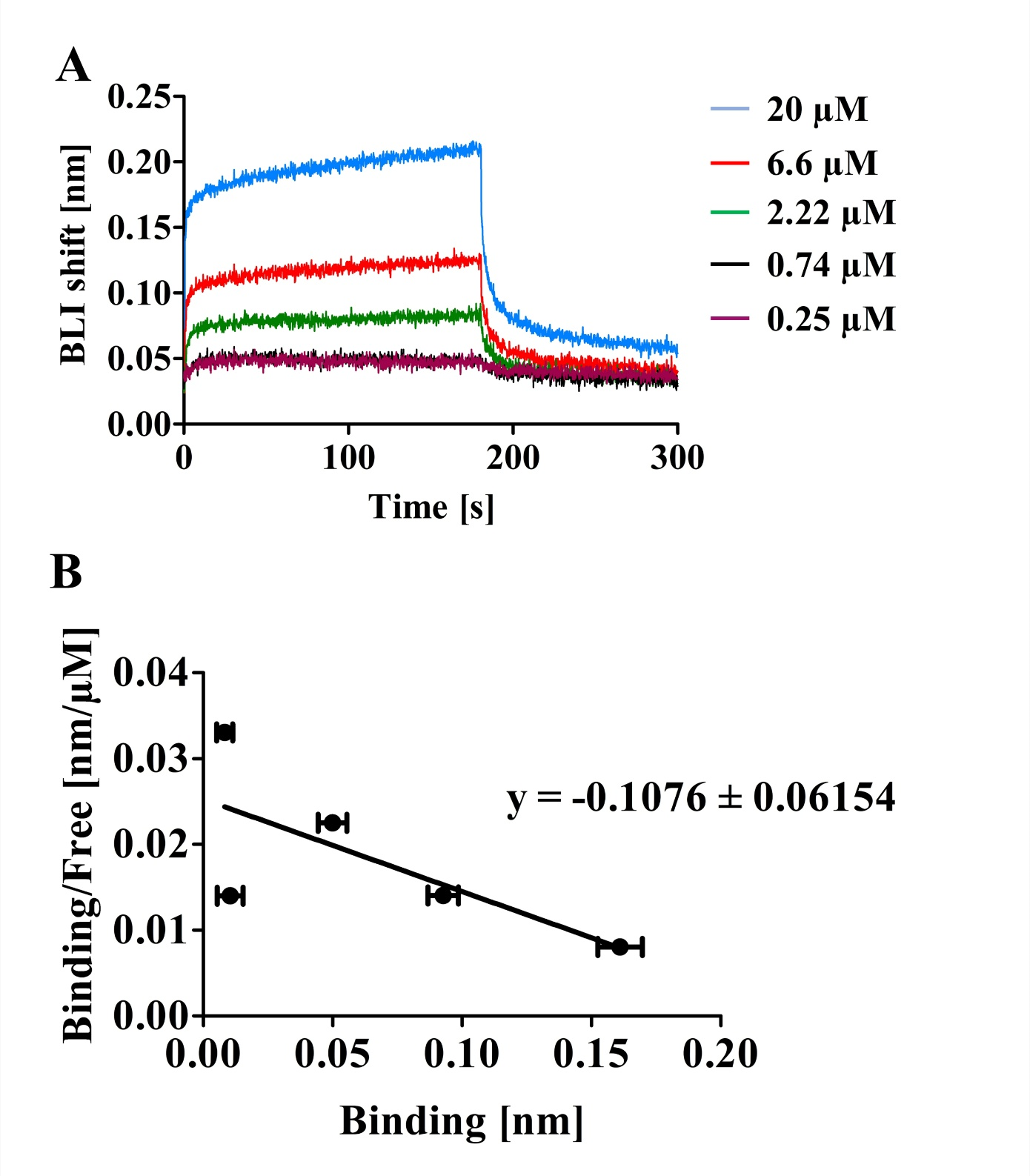

Supplement: S4 Fig — (A) Measurement performed using Octet AR2G biosensors. Pantinin-1 was tested at six concentrations (0–20 µM). The sensorgram includes a 180-second association phase followed by a 600-second dissociation phase at room temperature. (B) Scatchard plot of pantinin-1 binding to CHIKV nsP2pro based on BLI response data. Binding values at five concentrations were extracted from sensorgrams and plotted as Bound/Free versus Bound. The data are shown as mean ± SD (n = 3 technical replicates). The dissociation constant (KD) was calculated from the slope of the linear regression using the equation (KD) = –1/slope [1] yielding a (KD) of 9.29 µM. (TIF) [file pone.0346930.s004.tif]

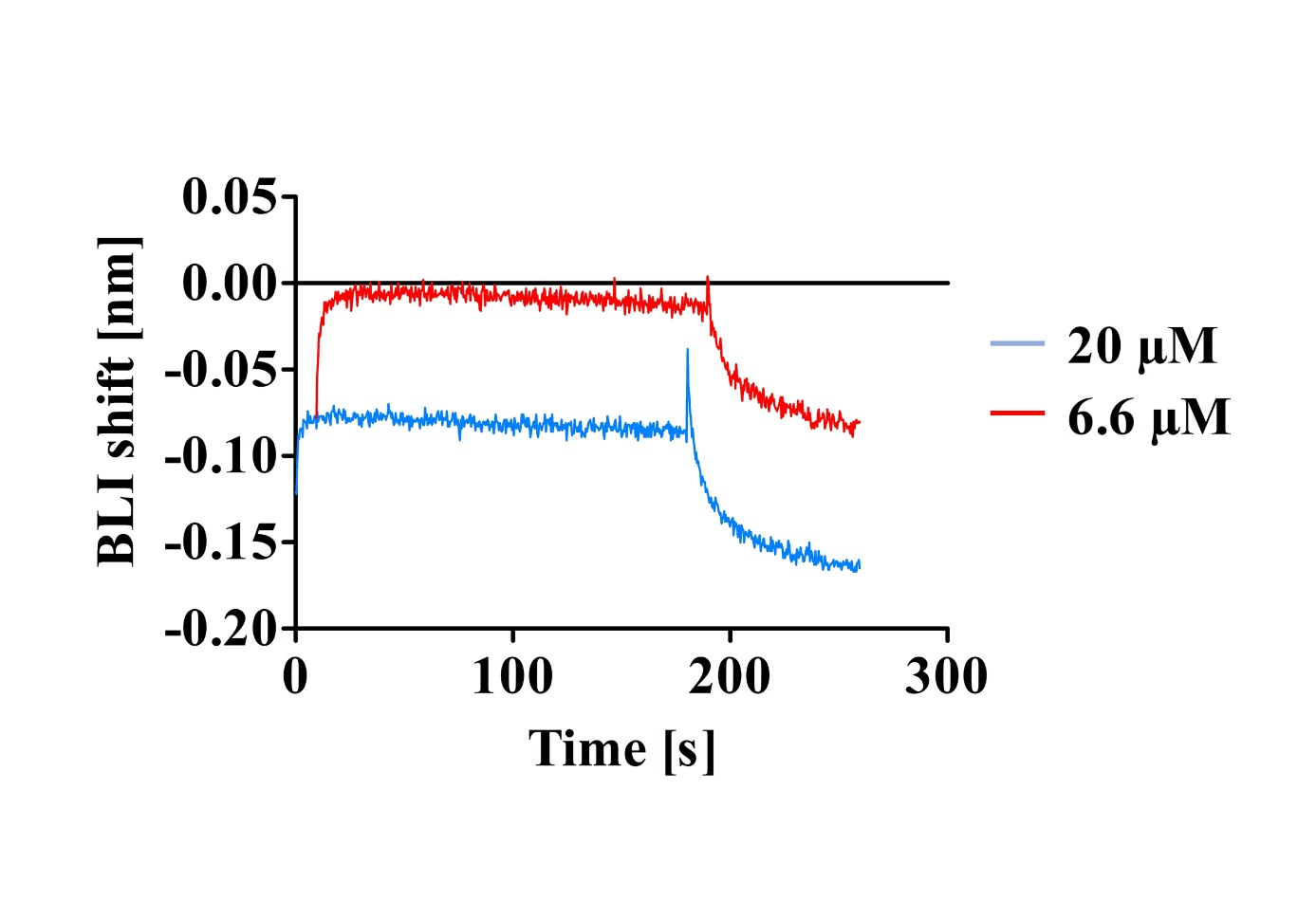

Supplement: S5 Fig — Measurements were performed using Octet AR2G biosensors without immobilized CHIKV nsP2pro to assess non-specific interaction with the sensor surface. Pantinin-1 was tested at two concentrations (20 µM and 6.66 µM). Each sensorgram includes a 180-second association phase and a 600-second dissociation phase at room temperature. No measurable binding response was detected at either concentration, indicating that pantinin-1 does not interact with the biosensor surface in the absence of the target protein. (TIF) [file pone.0346930.s005.tif]

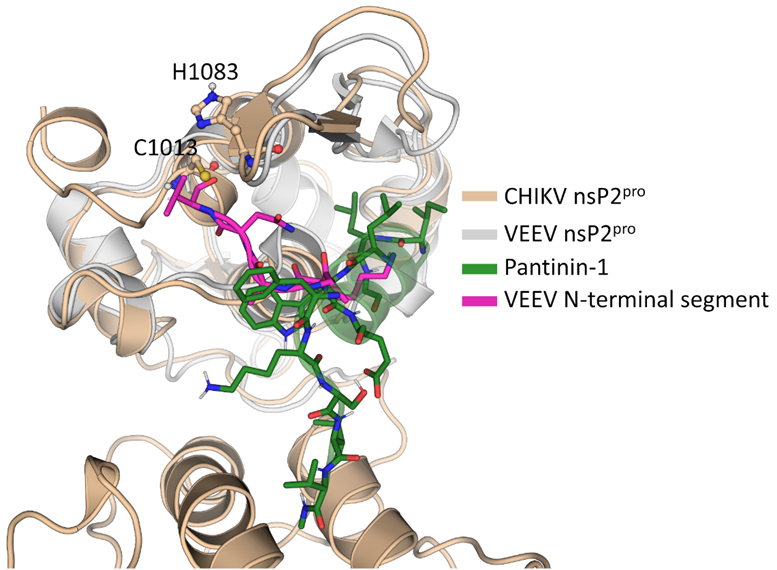

Supplement: S6 Fig — The figure showing an N-terminal segment inserted into the active site in a substrate-like conformation (PDB ID: 8DUF). (TIF) [file pone.0346930.s006.tif]
