# Supplementary material for: Inhibition of Chikungunya virus nsP2 protease in vitro by scorpion venom peptide pantinin-1
Source: PLoS One. 2026 Apr 9;21(4):e0346930. doi: 10.1371/journal.pone.0346930 (PMC13065029; doi:10.1371/journal.pone.0346930)
Supplement: S1 Table — (DOCX) [file pone.0346930.s007.docx]

**Table S1.** Top-5 docking poses for pantinin-1 predicted by three docking algorithms along with their backbone RMSD values relative to Galaxy TongDock pose 1.

| **Docking program** | **Docking Pose** | **RMSD (Å)** |
| --- | --- | --- |
| Galaxy TongDock | **1*** | 0 |
|  | 2 | 2.43 |
|  | **3** | 8.33 |
|  | 4 | 11.36 |
|  | **5** | 12.60 |
| ClusPro | **0** | 35.52 |
|  | 1 | 37.04 |
|  | 2 | 36.59 |
|  | 3 | 6.61 |
|  | **4** | 13.01 |
| HDock | **1** | 9.78 |
|  | 2 | 6.98 |
|  | 3 | 8.67 |
|  | 4 | 36.22 |
|  | 5 | 1.88 |

*Poses selected for MD refinement are written in bold.
