# Supplementary material for: Inhibition of Chikungunya virus nsP2 protease in vitro by scorpion venom peptide pantinin-1
Source: PLoS One. 2026 Apr 9;21(4):e0346930. doi: 10.1371/journal.pone.0346930 (PMC13065029; doi:10.1371/journal.pone.0346930)
Supplement: Attachment — Submitted filename: Mastalipour et al. PLOS ONE_Response to the reviewer.pdf [file pone.0346930.s009.pdf]

January 9th, 2026

Seth Agyei Domfeh, PhD

Academic Editor

PLOS ONE

Manuscript ID: **PONE-D-25-43080**

Dear Dr. Domfeh,

we are submitting the revised manuscript "**Inhibition of Chikungunya virus nsP2 protease *in vitro* by scorpion venom peptide pantinin-1**" by Mastalipour et al.

We have replied to all comments raised by the reviewers. Enclose you can find the response and the changes made by the authors.

We hope our manuscript matches the criteria to be published in *PLOS ONE* and we believe that this article will highly attract the readers of the Journal.

Thank you for your consideration!

Sincerely,

Dr. Raphael J. Eberle
Institute of Biochemical Plant Physiology
Heinrich-Heine-University Düsseldorf
Universitätsstraße 40225 Düsseldorf, Germany
Email: eberler@hhu.de
Phone: +49 211 - 811 15232

Response to the Reviewer

Reviewer #1

This study investigates the inhibitory activity of pantinin-1, a venom-derived antimicrobial peptide from Pandinus imperator, against the nonstructural protein 2 protease (nsP2pro) of Chikungunya virus (CHIKV), a key enzyme in viral replication. Using a FRET-based assay, pantinin-1 was shown to inhibit nsP2pro in vitro with an IC_{50} of 6.4 μM. Kinetic analysis confirmed a competitive inhibition mechanism, while biolayer interferometry determined a dissociation constant (KD) of 9.29 μM. Cytotoxicity assays in HEK 293 and Vero cells revealed no toxicity up to 20 μM, with moderate effects at 40 μM and higher. In silico molecular docking and molecular dynamics simulations supported binding at the protease active site, identifying key hydrophobic residues (L3, W7, F10, I13, V14) and substrate-binding residues (Y1079, Y1047, W1084) as critical for interaction.

The results demonstrate that pantinin-1 exerts potent inhibitory activity against CHIKV nsP2pro within a concentration range below the cytotoxic threshold. These findings suggest that pantinin-1 may serve as a lead compound for developing antiviral agents against CHIKV. As the authors noted, further investigations, particularly in virus-infected cell models, are required to validate its antiviral activity. The experiments are properly designed and executed, and the conclusions are convincingly supported by the data. As a reviewer, I have no critical comments on the manuscript and recommend its publication in PLOS One.

Response 1

Thank you for reviewing and evaluating our manuscript.

Reviewer #2

The manuscript titled "Inhibition of Chikungunya virus nsP2 protease in vitro by scorpion venom peptide pantinin-1" investigates the antiviral potential of pantinin-1, a 14-amino-acid antimicrobial peptide derived from Pandinus imperator. Using a series of biochemical and biophysical assays, the authors demonstrate that pantinin-1 inhibits CHIKV nsP2 protease activity with an IC_{50} of 6.4 ± 2.04 μM and shows competitive inhibition behaviour. Biolayer interferometry reveals a KD of ~9 μM, suggesting direct binding to nsP2pro. Cytotoxicity assays show no major toxicity up to 20 μM. Molecular docking and MD simulations identify a putative binding mode at the substrate-binding pocket. Overall, the manuscript presents a novel venom-derived peptide as a promising nsP2pro inhibitor and provides a mix of experimental and computational evidence supporting its potential as an antiviral lead.

Major Comments

Comment 1

The concept of targeting nsP2 protease is well established, and the authors correctly highlight the scarcity of potent inhibitors. Demonstrating inhibition by pantinin-1 is novel and relevant. However, the manuscript would benefit from a clearer articulation of how pantinin-1 advances the field beyond prior peptide inhibitors (e.g., P1 peptide, RA-0002034). At present, the novelty seems incremental.

Response 1

We thank the reviewer for this important observation and agree that nsP2 protease is a well-validated antiviral target. While previous studies have reported peptide-based inhibitors such as P1 and small molecules including RA-0002034, pantinin-1 advances the field in several meaningful ways. First, pantinin-1 is a naturally occurring venom-derived peptide, representing a distinct and underexplored chemical space compared with synthetic peptides or repurposed compounds previously described. Importantly, it is a short 14–amino acid peptide, considerably smaller than earlier peptide inhibitors, which offers practical advantages for synthesis, structural optimization, and structure–activity relationship studies.

Beyond its origin and size, our work positions pantinin-1 as a new lead scaffold rather than a standalone inhibitor. Venom-derived peptides are biologically evolved molecules that have historically served as successful starting points for drug development, and their application to CHIKV nsP2 protease has not been previously reported. In addition, our study provides an integrated biochemical and biophysical characterization, including enzymatic inhibition, competitive binding kinetics (KD), cytotoxicity profiling, and *in silico* interaction analysis. This comprehensive approach strengthens the mechanistic understanding of $nsP2^{pro}$ inhibition and establishes a clear framework for rational optimization of pantinin-1 derivatives with improved potency and reduced toxicity.

Taken together, the novelty of this study lies not only in demonstrating nsP2 protease inhibition, but in introducing a venom-derived peptide scaffold with favorable size and tractability, thereby expanding the antiviral discovery landscape and providing a platform for future development of CHIKV therapeutics.

We included the following text in the manuscript **introduction**, "While nsP2 protease inhibitors have been previously described, pantinin-1 advances the field by introducing a venom-derived peptide from an unexplored chemical space. Its compact size, defined binding mode, and inhibitory activity within a non-cytotoxic concentration range position pantinin-1 as a promising starting point for further antiviral optimization."

Comment 2

A major limitation is the absence of direct antiviral assays (e.g., CHIKV infection in Vero/BHK cells). Since cytotoxicity data are available, inclusion of EC_{50} values (or at least plaque-reduction and viral

RNA quantification) would substantially strengthen the conclusions. Without this, it is difficult to evaluate whether the protease inhibition observed *in vitro* translates into antiviral activity.

Response 2

We thank the reviewer for this important and well-taken comment. We fully agree that direct antiviral assays, such as CHIKV infection studies in Vero or BHK cells and determination of EC_{50} values, would substantially strengthen the translational relevance of our findings. The primary objective of the present study, however, was to establish proof-of-concept biochemical validation of pantinin-1 as an inhibitor of CHIKV nsP2 protease and to characterize its mechanism of action through enzymatic, kinetic, binding, and *in silico* analyses.

While cytotoxicity data demonstrate that pantinin-1 inhibits $nsP2^{pro}$ at concentrations below that causing significant cell toxicity, we acknowledge that enzymatic inhibition does not necessarily translate directly into antiviral efficacy in cell-based systems. Further optimization of the pantinin-1 sequence and evaluation of antiviral activity, including plaque-reduction assays and viral RNA quantification, is therefore a critical next step and is currently planned as part of our ongoing work. We have revised the manuscript to explicitly acknowledge this limitation and to frame pantinin-1 as a lead scaffold rather than a validated antiviral agent, emphasizing the need for future cell-based and *in vivo* studies to confirm its antiviral potential.

We added therefore the following text to the **conclusion and outlook section**, "The present work establishes pantinin-1 as a biochemical inhibitor of CHIKV nsP2 protease. After further optimization of the peptide, the determination of EC_{50} values using plaque-reduction and viral RNA assays will be essential to confirm its potential as an antiviral lead."

Comment 3

The Lineweaver–Burk analysis suggests competitive inhibition, but these double-reciprocal plots are prone to error and are no longer recommended as standalone mechanistic proof. Consider supplementing with nonlinear global fitting of Michaelis–Menten data/enzyme progress curve analysis.

Response 3

Thank you for the suggestion, the lineweaver-burk analysis is to see in combination with our docking and MD approach. In both cases the results show, that Pantinin-1 act as a competitive inhibitor. Especially the docking and MD approach demonstrated, that the peptide bind near or within the substrate-binding site, which could block this region for the interaction with the substrate. An inhibitor that bind in this substrate-binding site stand in competition with the substrate and is therefore a competitive inhibitor. However, we added a new sub-figure (1C), in figure 1, showing the nonlinear global fitting of Michaelis-Menten enzyme/inhibitor progress curve. Which even shows the typical characteristics of a competitive inhibitor by increasing the Km value. Which is described for several inhibitors with a competitive inhibition mode, please have a look in the following publications:

- Lee et al. 2024. https://doi.org/10.3390/biom14010099
- Vazquez-Armenta et al. 2025. https://doi.org/10.3390/catal15030257
- Coria et al. 2016. https://doi.org/10.4049/jimmunol.1501188
- Venditti et al. 2013. https://doi.org/10.1021/cb400027q
- Sorribas, Guillén and Sosa 2019. https://doi.org/10.1007/s00424-018-2241-x

We revised the results section and the discussion section accordingly.

Comment 4

The MD simulations reveal stable poses, but the conclusion that pantinin-1 is a competitive inhibitor because it binds the active site is not fully convincing. The final MD pose appears shifted from the catalytic dyad, raising questions about how substrate exclusion occurs mechanistically. The authors should explicitly compare the peptide binding pose with the native substrate (P1234) cleavage motif or model the peptide and substrate simultaneously. Per-residue decomposition identifies strong hydrophobic contributions, but experimental validation (e.g., alanine-scanning) is essential before stating these as "key binding residues."

Response 4

We are grateful for this comment, as mentioned in response 3, the docking and MD approach demonstrated, that pantinin-1 bind near or within the substrate-binding site, which block this region for the interaction with the substrate. However, the predicted binding mode of pantinin-1, although leaving the catalytic residues exposed, hinders the binding of a substrate peptide at the Pn side. This result is presented in the SI of the current version (S6 Fig). In addition, we have added the following sentences to the manuscript results section:

"However, the predicted binding mode is still capable of sterically hindering substrate access to the active site. This is supported by the superposition of nsP2pro in complex with pantinin-1 and the crystal structure of the Venezuelan equine encephalitis virus (VEEV) nsP2pro containing an N-terminal segment in a substrate-like conformation within the active site (S6 Fig)."

Regarding the per-residue decomposition results, we have clearly stated in the manuscript that the predicted free energy contribution values are a prediction based on the proposed binding mode of pantinin-1. It is beyond the scope of the manuscript to perform alanine scanning to validate the predictions.

Comment 5

Although the peptide is non-toxic up to 20 μM, its IC50 is 6.4 μM, leaving a narrow therapeutic window. The discussion should compare this with the typical requirements for peptide antiviral drugs and address potential strategies to reduce cytotoxicity (e.g., peptide engineering, truncation, amino acid modification, etc.).

Response 5

We thank the reviewer for highlighting this important point. We acknowledge that the current IC_{50} value of pantinin-1 (6.4 μM) relative to its non-toxic concentration range suggests a modest therapeutic window, which is not uncommon for early-stage peptide leads. Importantly, many peptide antivirals undergo substantial optimization to improve selectivity and reduce cytotoxicity, and pantinin-1's short length and defined structure make it well suited for such engineering strategies, including residue substitution, charge modulation, truncation, and incorporation of non-natural amino acids. We have revised the Discussion to place our findings in the context of peptide antiviral development and to explicitly outline these optimization approaches.

We added the following text to the discussion section,

"Peptide-based antiviral leads often exhibit low micromolar inhibitory activity at early stages of development, frequently accompanied by a relatively narrow therapeutic window that necessitates subsequent optimization [71-73]. The amphipathic and α-helical nature of pantinin-1, while contributing to its bioactivity, may also underlie the observed cytotoxicity at higher concentrations, a characteristic commonly reported for membrane-active and venom-derived peptides [74–76]. Importantly, such limitations can be mitigated through peptide engineering strategies, including sequence truncation, charge redistribution, reduction of hydrophobicity, and incorporation of non-natural or D-amino acids to improve selectivity and safety profiles [77-79]. Given its short length and defined structure, pantinin-1 represents a suitable scaffold for rational optimization aimed at enhancing its therapeutic index. Similar optimization approaches have successfully advanced other peptide antivirals from micromolar leads to clinically relevant candidates [80,81]."

Comment 6

Protease resistance, serum stability, haemolytic activity, and degradation half-life are crucial for peptide therapeutics. The manuscript should at least discuss existing data on pantinin-1 stability and susceptibility to proteolytic degradation.

Response 6

We added the following information to the manuscript discussion section.

"Pantinin-1 and -2, isolated from the scorpion *Pandinus imperator*, are known to have activity against Gram-positive bacteria, fungi, and viruses. Beside this broad antimicrobial impact, it was shown that Pantinin-1 displays substantial stability in human serum and a strong resistance to proteolytic degradation. After 16 hours of incubation, approximately 70–80% of the peptide remained intact. Furthermore, Pantinin-1 caused no significant hemolysis of human erythrocytes at concentrations up to 512 μg/mL. While these results indicate the potential for an extended biological half-life, detailed *in vivo* pharmacokinetic data have yet to be reported [56-59]."

- Giugliano et al. 2025. https://doi.org/10.3389/fmicb.2025.1569719
- Giugliano et al. 2025. https://doi.org/10.3390/pathogens14070713
- Giugliano et al. 2025. https://doi.org/10.1002/cmdc.202500333

- Capasso et al. 2025. https://doi.org/10.3390/microorganisms14010068

Comment 7

Result 3.1: As this result represents the inhibitory effect of pantinin-1 on purified CHIKV nsP2 protease. The main image showing the purified nsP2 protease is missing. Provide an image of the purified nsP2 protease (Coomassie gel image).

Response 7

We thank the reviewer for bringing up this important point. We have now added an image of a Coomassie stained SDS PAGE of purified nsP2 protease. The image has been included in the supplementary material (S1 Fig.).

Comment 8

Figure 1A: It would be better to provide the confidence score of the alpha fold predicted image of Pantinin-1. Figure 1: 1B- Only the inhibitory effect of the compound is mentioned in the result section 3.1. whereas in Figure 1 B, the percentage of activity and inhibition both are depicted. Provide the detailed methods and results for the calculation of the activity percentage.

Figure 1c: Describe the methodology of IC50 in the Materials and Methods section.

Response 8

We included the AlphaFold confidence value of the predicted structure (>90%) in Fig. 1.

Comment 9

Figure 2: The Author has mentioned N.control in both Figure 2A and 2B, but has not explained about this N.control, either in the result part or in the figure legend. In the figure legend, Triton X-100 was used as a positive control. Please specify the positive and negative controls and include them in the figure, the figure legend, and the respective results section.

Response 9

Thank you for the comment; we changed N. control to Triton X-100 in Figure 2 accordingly and uploaded the revised figure.

Comment 10

The Author has mentioned that Pantinin-1 shows an inhibitory effect on the nsP2 protease by purifying the protease. To demonstrate its advantage in CHIKV viral replication, the author should assess antiviral activity in the presence of pantinin-1.

Response 10

Thank you for the comment, but as already mentioned in the response to the main comment 2, we agree with the reviewer, that direct antiviral assays, including CHIKV infection experiments in Vero or BHK cells and determination of EC_{50} values, would considerably enhance the translational significance of this work (This experiments are planned). Nonetheless, this study was designed to establish biochemical proof of concept for pantinin-1 as an inhibitor of the CHIKV nsP2 protease and to characterize its mechanism of action through enzymatic, kinetic, binding, and *in silico* analyses.

Further optimization of the pantinin-1 sequence and evaluation of its antiviral activity, including plaque-reduction assays and viral RNA quantification, are essential next steps and are planned as part of our ongoing work. In the revised manuscript, we explicitly acknowledge this limitation and present pantinin-1 as a lead scaffold rather than a validated antiviral compound, highlighting the necessity of future cell-based and *in vivo* validation.

We added therefore the following text to the conclusion and outlook section, "The present work establishes pantinin-1 as a biochemical inhibitor of CHIKV nsP2 protease. After further optimization of the peptide, the determination of EC_{50} values using plaque-reduction and viral RNA assays will be essential to confirm its potential as an antiviral lead."

Comment 11

Methodology 2.7: The Author has selected the default parameter, but the parameter used in this study has not been explained properly.

Response 11

Thank you for this pertinent comment; however, it is not straightforward to address. The docking simulations were performed using the default settings implemented in the respective docking web servers. Detailed descriptions of the algorithms and parameters employed under default conditions are provided in the documentation available on each server, to which the reader is referred.

Comment 12

Figures indicate triplicates, but it is unclear if these were biological or technical replicates. Provide replicates clearly and specify how many independent experiments were performed.

Response 12

We included the missing information accordingly.

Minor Comments

Comment 1

The manuscript is mostly well written but would benefit from English editing. Several grammatical and typographical errors occur, e.g.: “Venom-derived peptides are new approach…” → “are a new approach”, “inhibition effect of the pantinin-1” → “inhibitory effect of pantinin-1”.

Response 1

We revised the manuscript accordingly.

Comment 2

Figure legends should specify statistical tests, sample numbers (n), and p-values (wherever applicable).

Response 2

We added the missing information accordingly.

Comment 3

Figure 1A (AlphaFold3 structure) should include confidence scores (pLDDT).

Response 3

We included the confidence sore accordingly.

Comment 4

Specify whether nsP2pro was active-site verified (e.g., via autocleavage assay).

Response 4

Yes, the nsP2^{pro} active site of different alpha viruses was verified previously, especially for Chikungunya Virus (CHIKV) and Venezuelan Equine Encephalitis Virus (VEEV), confirming its catalytic Cysteine (C478 in CHIKV) and defining its papain-like mechanism, substrate specificity (P1-P1' cleavage), and conformational dynamics using crystal structures, mutagenesis, mass spectrometry, and molecular dynamics. Please have a look at the following publications:

- Narwal et al. 2018. https://doi.org/10.1016/j.ijbiomac.2018.05.007
- Li et al. 2025. https://doi.org/10.1038/s41598-025-14697-3
- Merten et al. 2024. https://doi.org/10.1073/pnas.2409166121
- Ghoshal et al. 2025. https://doi.org/10.1038/s41598-025-91673-x

Comment 5

Clarify DMSO final concentration in enzyme assays; enzymatic activity can be sensitive.

Response 5

Thank you for the comment; we included the results of a DMSO test in which concentrations of 0, 0.5, 1, 2, 4, 6, 12 and 20% DMSO were tested. Up to 6 % DMSO we observed protease activity close to 100 %. We planned and performed the experiments using maximum DMSO concentration of 2% in the assay solution containing the protease. The result of the DMSO test have been added to the supplementary material, S2 Fig.

Comment 6

Provide RMSD plots in the supplementary data for all poses.

Response 6

Actually, RMSD plots for all the simulated docking poses were already provided in Figure 3. Nonetheless, we have included a supplementary table (S1 Table) with the RMSD values of all docking poses in an attempt to respond to this comment.

Comment 7

Include docking scores or binding energy ranks before MD refinement to show initial selection criteria.

Response 7

The criterion used to select docking poses was their ranking, as established independently by each docking algorithm. As described in Methodology 2.7, the top-ranked pose produced by each docking program, together with three additional poses displaying high conformational diversity (i.e., large pair-wise peptide RMSD values) among the top-five solutions, were selected for subsequent MD refinement. Figure 3 reports the pose numbers corresponding to the ranking positions assigned by each docking program. Importantly, because ClusPro, GalaxyTongDock, and HDOCK rely on distinct and non-comparable scoring functions, the absolute docking scores themselves are not directly interpretable across methods. In this context, pose ranking provides a more robust and method-consistent criterion, allowing the identification of the most representative and diverse binding modes within each docking framework.

Comment 8

Ensure consistency in journal name abbreviations.

Response 8

We revised the journal name abbreviations accordingly.

Comment 9

Table 3: Add a column indicating the experimental method used to assess inhibition (e.g., MST, FRET, cell-based).

Response 9

Thank you for the suggestion, we added the experimental methods accordingly.
