# Supplementary material for: Inhibition of Chikungunya virus nsP2 protease in vitro by scorpion venom peptide pantinin-1
Source: PLoS One. 2026 Apr 9;21(4):e0346930. doi: 10.1371/journal.pone.0346930 (PMC13065029; doi:10.1371/journal.pone.0346930)
Supplement: Attachment — Submitted filename: Mastalipour_et_al._PLOS_ONE_Response_to_the_reviewer_auresp_2.pdf [file pone.0346930.s010.pdf]

February 27th, 2026

Seth Agyei Domfeh, PhD

Academic Editor

PLOS ONE

Manuscript ID: **PONE-D-25-43080R1**

Dear Dr. Domfeh,

we are submitting the revised manuscript "**Inhibition of Chikungunya virus nsP2 protease *in vitro* by scorpion venom peptide pantinin-1**" by Mastalipour et al.

We have replied to the major comments raised by the reviewer. Enclose you can find the response and the changes made by the authors.

We hope our manuscript matches the criteria to be published in *PLOS ONE* and we believe that this article will highly attract the readers of the Journal.

Thank you for your consideration!

Sincerely,

Dr. Raphael J. Eberle
Institute of Biochemical Plant Physiology
Heinrich-Heine-University Düsseldorf
Universitätsstraße 40225 Düsseldorf, Germany
Email: eberler@hhu.de
Phone: +49 211 - 811 15232

Response to the Reviewer

<u>Reviewer #1</u>

I have no critical comments on the manuscript. The study is well designed, clearly presented, and the results are sound and adequately discussed; therefore, I recommend its publication in PLOS One.

Response Reviewer#1

Thank you for reviewing and evaluating our manuscript.

<u>Reviewer #2</u>

The responses submitted by the authors are satisfactory. However, CHIKV infection studies in at least Vero cells in the presence of pantinin-1 (including appropriate controls) and determination of EC_{50} values would substantially boost the translational relevance of the findings. It would definitely add more value to the manuscript.

<u>Response Reviewer#2</u>

We sincerely thank the reviewer for this thoughtful and constructive comment. We fully agree that *in vitro* infection studies involving CHIKV-infected cell lines would substantially enhance the translational value of the manuscript.

At this stage, however, we respectfully decline to include CHIKV infection experiments. The primary objective of the present study was to identify and characterize a lead peptide candidate and determine its initial in vitro activity (IC_{50}) under controlled conditions. We consider this an essential first step in a structured drug development process.

Importantly, the current peptide still requires further optimization to improve potency, metabolic stability, cell uptake and overall pharmacological properties. We believe that performing infection-based experiments prior to sequence optimization would be premature and may not fully reflect the therapeutic potential of an improved candidate. Our immediate focus is therefore on rational peptide refinement to enhance efficacy. Once an optimized version is obtained, we fully agree that evaluation in CHIKV infection models—followed by in *vivo studies*—will be scientifically justified and necessary.

We would also like to emphasize the broader context of this work. Chikungunya virus is expanding geographically, and Europe itself is increasingly at risk of outbreaks. We do not claim to have developed a definitive therapeutic solution. Rather, we present a promising peptide scaffold that demonstrates measurable in vitro activity and represents a credible starting point for further

development. With continued optimization, this candidate could progress toward more advanced preclinical evaluation.

To address the reviewer's concern, we have clarified in the revised manuscript (Conclusion and Outlook section) that:

1. The current findings represent an early-stage lead identification effort.

2. Peptide optimization is ongoing.

3. Infection-based and in *vivo* studies are planned as future steps once an improved candidate is available.

We respectfully hope the reviewer and the editorial office will consider that the present manuscript provides a solid and scientifically sound foundation for subsequent translational studies. We sincerely appreciate the reviewer's valuable input, which has helped us better position the scope and future direction of our work.
